# Bioaccessibility and cellular transport study of silver and titanium dioxide nanoparticles from exposed seaweed and mussels using Caco-2 cells

**DOI:** 10.1007/s00604-025-07066-4

**Published:** 2025-03-08

**Authors:** Juan José López-Mayán, Raquel Domínguez-González, María Carmen Barciela-Alonso, Elena Peña-Vázquez, Antonio Moreda-Piñeiro, Pablo Taboada-Antelo, Pilar Bermejo-Barrera

**Affiliations:** 1 Trace Element, Spectroscopy and Speciation Group (GETEE), Instituto de Materiais (iMATUS), Faculty of Chemistry, University of Santiago de Compostela, Av. das Ciencias, s/n, 15782 Santiago de Compostela, Spain; 2 Colloids and Polymer Physics Group, Instituto de Materiais (iMATUS), Department of Particle Physics, Faculty of Physics, University of Santiago de Compostela, Rúa Xosé María Súarez Núñez, s/n, E15782 Santiago de Compostela, Spain

**Keywords:** Bioaccessibility, Caco 2 cells, Cellular transport, Single-particle-ICP-MS, Single-cell-ICP-MS

## Abstract

Bioavailability studies on pollution pre-concentrator organisms such as algae and mussels are necessary to ensure food safety, particularly in the case of nanomaterials whose industrial applications have increased in recent years. Thus, the bioaccessibility and the bioavailability of total Ag and Ti and AgNPs and TiO_2_NPs from raw and cooked seaweed (*Palmaria palmata* and *Ulva* sp.) and cooked mussels (*Mytilus edulis*) exposed to 1.0 mg L^−1^ of 15 nm PVP-AgNPs, 1.0 mg L^−1^ of 25 nm citrate-TiO_2_NPs, or 1.0 mg L^−1^ 5 nm citrate-TiO_2_NPs, for 28 days, were evaluated. Samples were subjected to an in vitro process simulating human gastrointestinal digestion while cell transport from the intestinal lumen to the bloodstream was mimicked using Caco-2 cell cultures. Total Ag and Ti in the digest samples, the bioaccessible fractions, the apical, and basolateral fractions were determined by ICP-MS, while AgNPs and TiO_2_NPs were determined by single-particle-ICP-MS (SP-ICP-MS). Finally, the presence of nanoparticles in the Caco-2 cells was verified by single-cell-ICP-MS (SC-ICP-MS). AgNP bioaccessibility ratios were between 22% and 97% for seaweed and 18% for mussels, while for TiO_2_NPs were between 17% and 81% (seaweed) and 76% and 100% (mussels). Nanoparticle cellular transports were in all cases less than 1%. However, the mass percentages of Ag as NPs and Ti as NPs in the Caco-2 cells for raw and cooked seaweed were 9% and 7% and 20% and 6%, respectively. These results confirm a small transport of the nanoparticles through the Caco-2 cells under the proposed experimental conditions.

**Graphical abstract:**

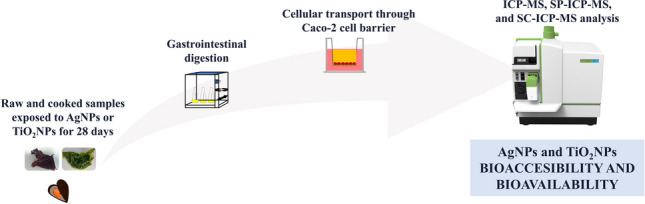

**Supplementary Information:**

The online version contains supplementary material available at 10.1007/s00604-025-07066-4.

## Introduction

According to the Nanotechnology Products Database, silver nanoparticles (AgNPs) and titanium dioxide nanoparticles (TiO_2_NPs) are one of the most commonly used nanoparticles in the industry due to their novel properties in comparison to their bulk materials [1]. The antiviral activity, antibacterial, antifungal, and anticancer properties of AgNPs [2, 3] make them widely used, mainly in medicine, textile, cosmetics, home appliances, environmental applications, construction, and food industry [1], while the photocatalytic activity of TiO_2_NPs is valuable in cosmetics, construction, textile, and renewable energy industries [1, 4]. The use of TiO_2_ as the food additive E-171 was banned by the European Food Safety Authority (EFSA) from February 2022 [5, 6].

The dispersed and extensive use of AgNPs and TiO_2_NPs causes them to be emitted into the environment, where marine organisms can interact with these nanomaterials [7]. Bivalve mollusks, as suspension filter feeders, are well-recognized sensitive bioindicators for biomonitoring the possible impacts of anthropogenic pollutants on the health of the aquatic environment [8]. Furthermore, seaweeds are used in bioremediation due to their ability to pre-concentrate heavy metals and emergent contaminants like NPs at concentrations a thousand times higher than in seawater [9].

The major route of entry of NPs into the body was found to be oral uptake due to the high levels of NPs in food, the large absorption area, and the relatively high translocation rate [10]. The ingested nanoparticles can cross the epithelium of the small and large intestines, reach the circulatory system, and consequently be distributed through several organs, while the non-absorbed nanoparticles are excreted by the feces [8, 11, 12]. Due to the growing number of modified NPs that can not all be tested in animal trials, in vitro models have drawn a lot of attention to study the bioaccessible and bioavailable fractions [10]. Several studies were found in the case of in vitro gastrointestinal digestion and transcellular transport assays using Caco-2 cells (human epithelial colorectal adenocarcinoma) for AgNPs [13–18] and TiO_2_NPs standards [19–23].

Regarding AgNPs, Kämpfer et al. [18] compared the response to AgNO_3_ and AgNPs of a simulated healthy and inflamed intestine using co-cultures of Caco-2/THP-1 cells [18]. Salman et al. [24] reported that AgNPs decreased the viability of Caco-2 cells in a dose- and time-dependent manner inducing apoptosis [24]. Xu et al. [25] used cell line models (Caco-2 and HIEC-6) and concluded that polyethyleneimine-AgNPs induced toxicity (oxidative stress, mitochondrial impairment, and release of inflammatory cytokines) and reduced the intestinal barrier function [25].

On the other hand, Tada-Oikawa et al. [26] studied the effects of different TiO_2_NP crystal structures on Caco-2 cells. Results showed that anatase TiO_2_NPs induced an inflammatory response, and the high concentrations of anatase, rutile, and P25 TiO_2_NPs reduced the cellular viability in Caco-2 cells [26]. Gerloff et al. [27] studied the cytotoxicity, oxidative stress, and DNA damage of five different TiO_2_NP compositions, where anatase/rutile showed mild DNA damage in comparison to pure anatase powders [27]. Krüger et al. [28] also described the activation of inflammatory pathways in Caco-2 cells exposed to 5–10 nm TiO_2_NPs [28]. Furthermore, Vila et al. [29] reported a non-toxic effect in the range of 1–200 mg L^−1^ of TiO_2_NPs in the biomarkers analyzed. These authors observed that TiO_2_NPs uptake and translocation through the Caco-2 cells did not modify the integrity of the monolayer [29].

Although Taboada-López et al. [8] studied the presence of AgNPs and TiO_2_NPs in raw mollusk samples and their bioaccessibility and bioavailability through Caco-2 cell monolayers, no reports were found on seaweed and marine samples submitted to bioaccumulation assays of PVP-AgNPs and citrate-stabilized TiO_2_NPs. Thus, the main objectives of this study are the evaluation of total Ag, total Ti, AgNP, and TiO_2_NP bioaccessibility and bioavailability with cellular transport through a Caco-2 cell monolayer in raw and cooked seaweed (*Palmaria palmata* and *Ulva* sp.), and in cooked mussels (*Mytilus edulis*) previously exposed to nanoparticles for 28 days. Total Ag and Ti and AgNPs and TiO_2_NPs were determined by ICP-MS and SP-ICP-MS, and the nanoparticle content in the Caco-2 monolayer was analyzed by SC-ICP-MS.

## Materials and methods

### Instrumentation

A NexION® 2000 inductively coupled plasma mass spectrometer from Perkin Elmer (Waltham, MA, USA) was used for Ag and Ti determination. The ICP-MS working in single-particle mode (SP-ICP-MS) with the Syngistix™ Nano Application 2.5 software was used for the determination of AgNP and TiO_2_NP content and size distribution. The single-cell Micro DX autosampler from PerkinElmer with the Asperon™ spray chamber allows to work in single-cell mode (SC-ICP-MS), with the Syngistix™ Single Cell Application Software, for the determination of AgNPs and TiO_2_NPs internalized in cells. Other instrumentation used in this study is included in the Supplementary information.

### Standards, reagents, and materials

All the details about standards, reagents, and materials are also provided in the Supplementary information.

### Seaweed and mussel samples

**Fig. 1:**
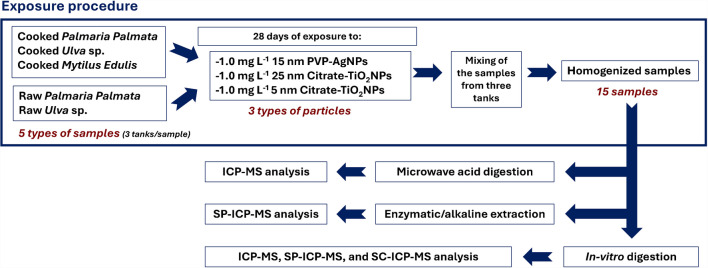
Scheme of the exposure procedure and the analytical treatment

Seaweed (*Palmaria palmata* and *Ulva* sp.) and mussels (*Mytilus edulis*) used for bioaccessibility and cellular transport experiments were cultured at Indigo Rock Marine Research Center (Cork, Ireland). The study was carried out with specimen samples exposed to 1.0 mg L^−1^ of 15 nm PVP-AgNPs or 1.0 mg L^−1^ citrate-coated TiO_2_NPs (25 nm or 5 nm), for 28 days with three replicate tanks per type of particle. The characterization of the materials has been carried out in previous research, and details are included in the Supplementary information. Exposure at a lower concentration (0.1 mg L^−1^) resulted in less bioaccumulation of both types of nanoparticles, and therefore, 1.0 mg L^−1^ was used to facilitate analytical detection during the bioaccessibility and cell transport assays. Seaweed and mussels were sampled from the three different tanks, mixed and homogenized to obtain enough amount of sample for the experiments (pool of samples). In vitro digestions were carried out with raw and cooked seaweed and cooked mussels. The study was conducted with fifteen samples: three raw *Palmaria palmata* samples (one exposed to AgNPs, one exposed to 25 nm TiO_2_NPs, and one exposed to 5 nm TiO_2_NPs), three cooked *Palmaria palmata* samples, three raw *Ulva* sp. samples, three cooked *Ulva* sp. samples, and three cooked *Mytilus edulis* samples. The cooking procedure consisted of boiling approximately 10 g of sample in 600 mL of ultrapure water for 5 min. Finally, the cooking water was removed by gravity filtration and the samples were collected and stored in polypropylene tubes at − 18 °C until use. Figure 1 shows a scheme of the exposure procedure and the analytical treatment.

### Sample digestion and extraction procedures

For total Ag and Ti determination in seaweed and mussel samples, microwave-assisted acid digestion was employed before ICP-MS analysis using the program reported by López-Mayán et al. [30]. The acid digestions were performed in triplicate with two blanks per digestion batch. The digested samples were diluted with ultrapure water up to 25 mL and stored in polypropylene tubes until ICP-MS analysis.

The extraction procedures of AgNPs and TiO_2_NPs were previously developed in our research group. Enzymatic extractions with pancreatin/lipase mixtures were used for AgNP [31] and TiO_2_NP [32] separation from mollusk samples. An enzymatic extraction using Macerozyme® R-10 was used for AgNP separation from seaweed [30], due to its ability to break the vegetal cell wall. TiO_2_NPs were extracted from seaweed using an alkaline extraction with TMAH [33]. Table S1 (see supplementary information) shows the main conditions for nanoparticle extraction using the procedures abovementioned.

### In vitro digestion procedures

**Fig. 2:**
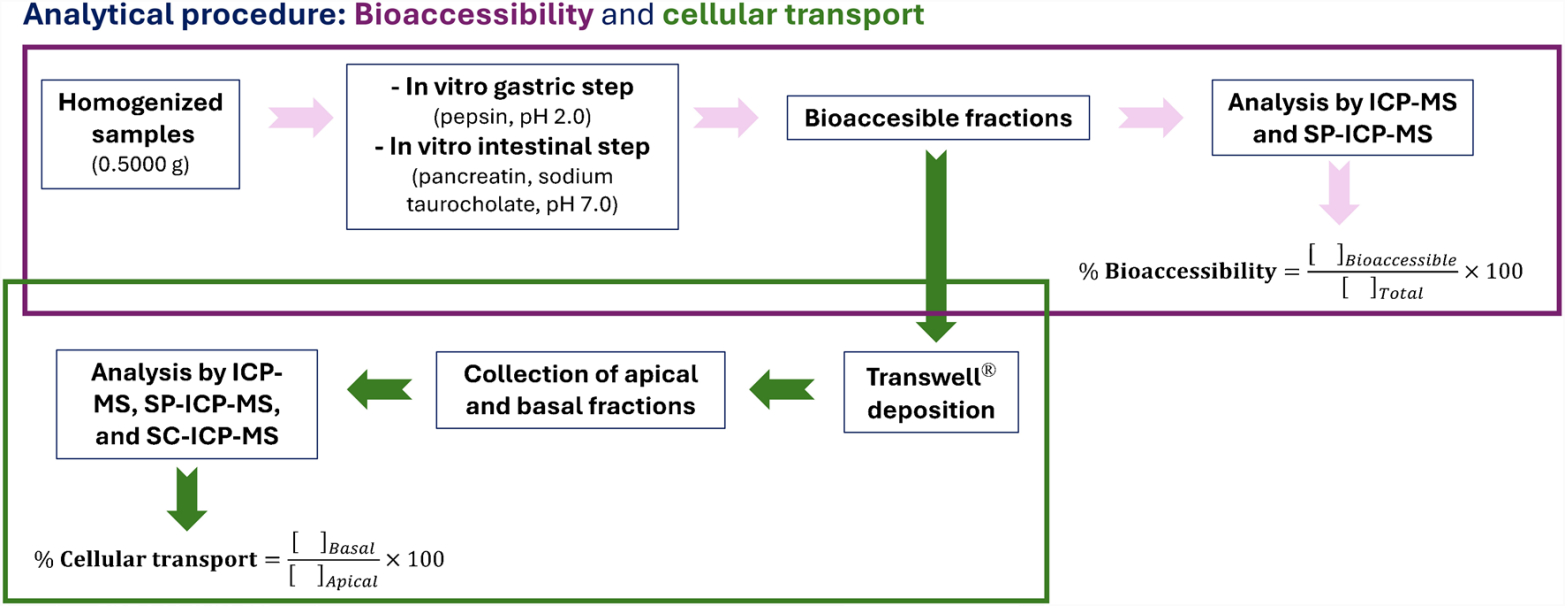
Scheme of the analytical procedure (bioaccessibility and cellular transport assays)

Each raw and cooked seaweed sample and each cooked mussel sample exposed to each type of particle (15 nm AgNPs, 25 nm TiO_2_NPs, or 5 nm TiO_2_NPs) were submitted in triplicate to the in vitro digestion and the cellular transport through the Caco-2 cell barrier. Figure 2 shows a scheme of the in vitro digestion procedures (bioaccessibility and cellular transport assays).

#### Bioaccessibility assay

The in vitro gastrointestinal digestion to obtain the bioaccessible fractions was performed following the procedure described elsewhere by Taboada et al. [8] with minor modifications (see Supplementary information). Briefly, a mass of 0.5000 g of samples (raw or cooked) were mixed during the gastric step (pH 2.0) with 10 mL of ultrapure water and 75 µL of gastric solution (160 g L^−1^ of pepsin in 0.1M HCl) and incubated for 2 h at 37 °C and 150 rpm. For the intestinal step (pH 7.0), a simulating solution (2.5 mL of 4 g L^−1^ of pancreatin and 2.58 g L^−1^ of sodium taurocholate in 0.1M of NaH_2_CO_3_) was added (incubation for 2 h). Three replicates of samples and two blanks were used for each digestion batch.

#### Cellular transport assay

All the details about the cellular transport assay (incubation conditions, formation of the Caco-2 cell monolayer, study of membrane integrity, etc.) are included in the Supplementary material.

Briefly, transport assays were performed adding 1.5 mL of the bioaccessible fraction into the apical compartment and 2 mL of HBSS in the basolateral chamber of the Transwell® and incubating for 1 h. The apical and basolateral fractions were collected and stored until their analysis by SP-ICP-MS. The cells were frozen at − 18 °C keeping their integrity using Cryopres dimethyl sulfoxide, until their analysis by SC-ICP-MS. Each bioaccessible fraction was submitted in triplicate to the cellular transport across the Caco-2 cell membrane with their respective blanks.

### Cell viability assay

The CCK-8 cytotoxicity kit was used to study the viability of the Caco-2 cells membrane after the addition of the bioaccessible fractions containing different salts from the in vitro intestinal digestion (bile salts or sodium taurocholate). The cell viability study was described in detail in the Supplementary information. Sodium taurocholate was selected for the in vitro intestinal *digestion* because it was biocompatible within the first two hours, which would be the maximum duration time allowed for the cellular transport experiments.

### Ag and Ti determination by ICP-MS

Total Ag and Ti concentrations were determined by ICP-MS. Supplementary information includes the operational conditions of ICP-MS (Table S2 (a)) and the limits of detection (LOD) and quantification (LOQ) (Table S3).

### AgNP and TiO_2_NP determination by SP-ICP-MS and SC-ICP-MS

AgNP and TiO_2_NP content and size distributions were determined by SP-ICP-MS. Supplementary information provides details about instrumental conditions (Table S2 (b)), calibration, sample dilutions, and LOD determination using Laborda et al. [34] criteria (Table S4). SC-ICP-MS was used to determine AgNPs and TiO_2_NPs internalized in the Caco-2 cells (see supplementary information and Table S2(c) for more data about conditions and LODs).

## Results and discussion

The bioaccessible fraction is considered the soluble fraction of a compound in the gastrointestinal medium available for absorption in the intestinal wall, whereas bioavailability (transport percentage ratio) refers to the fraction of a total dose of a substance ingested that arrives at the circulatory system of an organism [8]. The bioaccessibility ratio (in percentage) was calculated as the relation between the concentration in the bioaccessible fraction and the initial concentration in the sample. The transport percentage was calculated as the ratio between the concentration of an analyte in the basolateral compartment divided by the concentration added to the apical compartment of the Transwell® before the in vitro Caco-2 cell experiment (see Fig. 2).

### Total Ag and AgNP bioaccessibility

The results obtained in the total Ag and AgNP bioaccessibility study are shown in Table 1. Total Ag was determined in the digested samples and in the bioaccessible fractions after the gastrointestinal digestions of raw and cooked *Palmaria palmata*, *Ulva* sp., and cooked *Mytilus edulis*. The content of Ag in the digested samples was between 1.48 ± 0.23 and 3.66 ± 0.01 µg g^−1^ w.w. (wet weight) for cooked *Palmaria palmata*, and raw *Ulva* sp., respectively. The total Ag content in the bioaccessible fractions was between 0.29 ± 0.05 and 1.35 ± 0.08 µg g^−1^ w.w. for cooked *Palmaria palmata* and raw *Ulva* sp., respectively. *F*-test and *t*-tests were used to compare total Ag concentrations in the digests of raw and cooked seaweeds using Statgraphics Centurion XVIII. *P*-values in both cases were higher than 0.05, and therefore, no statistical differences were found between the mean Ag concentrations in raw and cooked seaweed. However, a decrease in total Ag concentration was found between the digested samples and the bioaccessible fractions for both kinds of seaweed (even in cooked and raw), resulting in bioaccessible ratios between 20 and 42% for raw *Palmaria palmata* and cooked *Ulva* sp., respectively. In the case of *Mytilus edulis*, total Ag concentration remained constant after the gastrointestinal digestion, resulting in bioaccessibility percentages of 100%.

**Table 1:** Total Ag and AgNP bioaccessibility ratios for seaweed and mussels

	Ag			AgNPs						
	Digests	Bioaccessible	B. ratio (%)	Extracts		Bioaccessible		Extracts	Bioaccessible	B. ratio (%)
	[Ag] (µg g^−1^)	[Ag] (µg g^−1^)		[AgNPs] (part g^−1^)	Size (nm)	[AgNPs] (part g^−1^)	Size (nm)	[Ag] as NPs (µg g^−1^)	[Ag] as NPs (µg g^−1^)	
Raw Palmaria	1.48 ± 0.23	0.29 ± 0.05	20	1.52 × 10^9^ ± 2.18 × 10^8^	23 ± 1	3.51 × 10^8^ ± 8.04 × 10^7^	27 ± 2	0.086 ± 0.021	0.035 ± 0.003	41
Cooked Palmaria	1.50 ± 0.33	0.36 ± 0.01	24	1.33 × 10^9^ ± 2.33 × 10^8^	22 ± 2	2.06 × 10^8^ ± 4.81 × 10^6^	34 ± 8	0.080 ± 0.001	0.018 ± 0.002	22
Raw Ulva	3.66 ± 0.01	1.35 ± 0.08	37	1.04 × 10^9^ ± 3.35 × 10^8^	29 ± 2	2.76 × 10^8^ ± 4.81 × 10^6^	34 ± 8	0.132 ± 0.019	0.083 ± 0.008	63
Cooked Ulva	2.56 ± 0.96	1.09 ± 0.13	42	2.46 × 10^8^ ± 1.14 × 10^8^	31 ± 1	2.55 × 10^8^ ± 9.06 × 10^7^	30 ± 1	0.039 ± 0.013	0.038 ± 0.011	97
Cooked Mytilus edulis	1.60 ± 0.50	1.65 ± 0.19	100	3.80 × 10^8^ ± 1.31 × 10^7^	39 ± 1	7.68 × 10^7^ ± 1.36 × 10^7^	37 ± 2	0.122 ± 0.003	0.022 ± 0.003	18

B. indicates “Bioaccessibility”

AgNP concentration was determined in the enzymatic extracts and in the bioaccessible fractions after gastrointestinal digestion. The mean NPs sizes were between 22 ± 2 and 31 ± 1 nm for seaweed extracts and between 27 ± 2 and 34 ± 8 nm for seaweed bioaccessible fractions. In the case of mussels, the mean AgNP sizes in the enzymatic extracts and the bioaccessible fractions were similar (39 ± 1 nm and 37 ± 2 nm, respectively).

Mass concentrations were calculated, taking into account: the mean sizes, AgNP concentration (part g^−1^), and assuming that AgNPs are spheric. Results in mass concentration (µg g^−1^ of Ag as NPs) in the extracts as well as the bioaccessible fraction are shown in Table 1. The bioaccessibility percentage ratios of Ag as NPs in seaweed ranged from 22 to 97% in the case of cooked *Palmaria palmata* and cooked *Ulva* sp. In cooked *Ulva* sp., 42% of the total Ag content was bioaccessible, while 97% of AgNPs were bioaccessible. However, in the case of cooked *Mytilus edulis*, although 100% of the Ag was bioaccessible, only 18% of the AgNPs in the sample were bioaccessible.

### Total Ti and TiO_2_NP bioaccessibility

The results obtained for the bioaccessibility assays of total Ti and TiO_2_NPs are shown in Table 2.

**Table 2:** Total Ti and TiO_2_NP bioaccessibility ratios for seaweed and mussels

	Ti			TiO_2_NPs						
	Digests	Bioaccessible	B ratio (%)	Extracts		Bioaccessible		Extracts	Bioaccessible	B. ratio (%)
	[Ti] (µg g^−1^)	[Ti] (µg g^−1^)		[TiO_2_NPs] (part g^−1^)	Size (nm)	[TiO_2_NPs] (part g^−1^)	Size (nm)	[Ti] as NPs (µg g^−1^)	[Ti] as NPs (µg g^−1^)	
Raw Palmaria (25 nm)	34.05 ± 3.59	9.94 ± 2.28	29	2.36 × 10^9^ ± 1.12 × 10^8^	86 ± 2	4.12 × 10^9^ ± 1.44 × 10^9^	67 ± 2	2.07 ± 0.32	1.52 ± 0.66	73
Cooked Palmaria (25 nm)	14.20 ± 1.91	4.61 ± 1.02	32	2.72 × 10^9^ ± 6.72 × 10^7^	86 ± 2	7.16 × 10^8^ ± 3.36 × 10^8^	77 ± 9	2.43 ± 0.03	0.40 ± 0.06	17
Raw Palmaria (5 nm)	5.73 ± 0.57	0.84 ± 0.01	15	7.47 × 10^8^ ± 2.06 × 10^8^	67 ± 5	-	-	0.28 ± 0.01	-	
Cooked Palmaria (5 nm)	1.78 ± 0.13	0.47 ± 0.05	26	-	-	-	-	-	-	
Raw Ulva (25 nm)	49.18 ± 3.87	28.06 ± 0.01	57	3.79 × 10^9^ ± 5.00 × 10^8^	80 ± 1	6.01 × 10^9^ ± 1.44 × 10^9^	65 ± 2	2.50 ± 0.17	2.02 ± 0.92	81
Cooked Ulva (25 nm)	19.03 ± 1.01	9.54 ± 0.73	50	3.39 × 10^9^	87 ± 1	1.57 × 10^9^ ± 1.23 × 10^8^	81 ± 2	2.97 ± 0.09	1.10 ± 0.16	37
Raw Ulva (5 nm)	47.50 ± 3.78	16.28 ± 0.65	34	4.69 × 10^9^ ± 5.12 × 10^8^	74 ± 7	3.09 × 10^9^ ± 8.25 × 10^8^	64 ± 2	2.50 ± 0.36	1.06 ± 0.23	42
Cooked Ulva (5 nm)	16.08 ± 1.62	12.48 ± 4.87	78	5.20 × 10^9^ ± 3.58 × 10^8^	62 ± 5	3.58 × 10^9^ ± 1.08 × 10^9^	57 ± 9	1.65 ± 0.51	0.82 ± 0.14	50
Cooked Mytilus (25 nm)	1.48 ± 0.22	1.52 ± 0.49	102	8.45 × 10^7^ ± 7.19 × 10^6^	96 ± 27	1.77 × 10^8^ ± 1.41 × 10^7^	67 ± 11	0.09 ± 0.01	0.10 ± 0.02	103
Cooked Mytilus (5 nm)	1.33 ± 0.02	1.89 ± 1.06	100	1.03 × 10^8^ ± 1.44 × 10^7^	78 ± 13	1.67 × 10^8^ ± 1.01 × 10^7^	76 ± 19	0.06 ± 0.02	0.05 ± 0.02	76

B. indicates “Bioaccessibility”;—indicates “ < LOD

Total Ti concentrations in seaweed digest samples were between 1.78 ± 0.13 and 49.18 ± 3.87 µg g^−1^ (w.w.) for cooked *Palmaria palmata* exposed to 5 nm TiO_2_NPs and for raw *Ulva* sp. exposed to 25 nm TiO_2_NPs, respectively. Significant differences between total Ti in cooked and raw *Palmaria* exposed to 25 nm or 5 nm TiO_2_NPs were observed (*P*-values < 0.05). A higher Ti concentration was found in *Palmaria palmata* exposed to 25 nm than to 5 nm TiO_2_NPs. Bioaccessibility percentages between 15% (for raw *Palmaria* exposed to 5 nm TiO_2_NPs) and 32% (for cooked *Palmaria* exposed to 25 nm TiO_2_NPs) were obtained. In the case of *Ulva* sp., a decrease in total Ti concentration was also found after cooking (*P*-value < 0.05). Ti concentration also decreased in the bioaccessible fractions compared to the digests in all kinds of seaweed. The bioaccessibility ratios were between 34 and 78% for raw and cooked *Ulva* sp. exposed to 5 nm TiO_2_NPs, respectively.

Total Ti contents in cooked mussels exposed to 25 and 5 nm TiO_2_NPs were statistically similar (*P*-value > 0.05) and the bioaccessible ratios resulted in both cases of 100%.

TiO_2_NPs were determined by SP-ICP-MS in the alkaline extracts of raw and cooked *Palmaria palmata* and *Ulva* sp., and in the enzymatic extracts of cooked *Mytilus edulis* exposed to these NPs (25 and 5 nm TiO_2_NPs) as well as in the bioaccessible fractions after gastrointestinal digestion.

The experimental mean sizes were higher than the nominal sizes of the NPs used to expose the samples (5 and 25 nm) probably due to the common aggregation phenomenon of TiO_2_NPs (see Supplementary information: characterization of PVP-AgNPs and TiO_2_-citrate NPs). The TiO_2_NP (part g^−1^) concentration was also transformed into mass of Ti (µg of Ti as NPs). The concentrations of Ti as NPs in alkaline extracts in *Palmaria palmata* were between 0.28 ± 0.01 and 2.43 ± 0.03 µg of Ti as NPs g^−1^ w.w. for raw *Palmaria* exposed to 5 nm TiO_2_NPs and cooked *Palmaria* exposed to 25 nm TiO_2_NPs, respectively. The content of Ti as NPs in the cooked *Palmaria palmata* exposed to 5 nm was lower than the LOD. No statistical differences between *Palmaria Palmata* before and after cooking were found for 25 nm. Ti as NPs was only detected in the bioaccessible fractions of cooked and raw *Palmaria* exposed to 25 nm TiO_2_NPs. The bioaccessibility percentages were 17% and 73% for cooked and raw *Palmaria* exposed to 25 nm TiO_2_NPs, respectively. On the other hand, Ti as NPs was quantified in all the alkaline extracts and bioaccessible fractions of *Ulva* sp., with contents between 1.65 ± 0.51 and 2.97 ± 0.09 µg of Ti as NPs g^−1^ w.w. in the alkaline extracts for cooked *Ulva* sp. exposed to 5 nm TiO_2_NPs and cooked *Ulva* sp. exposed to 25 nm TiO_2_NPs, respectively. A decrease in total Ti concentrations was observed after cooking but the concentration of TiO_2_NPs remained practically constant (*P*-values > 0.05). The concentration of Ti as NPs in the bioaccessible fraction of *Ulva* sp. decreased providing bioaccessibility percentages between 37 and 81% for cooked and raw *Ulva* sp*.,* both exposed to 25 nm TiO_2_NPs.

Regarding *Mytilus edulis*, the mass of Ti as NPs was between 0.06 ± 0.02 and 0.09 ± 0.01 µg of Ti as NPs g^−1^ w.w. of cooked mussels exposed to 5 and 25 nm, respectively, and no differences were found (*P*-value > 0.05). Bioaccessibility ratios of 76 to 100% were obtained for cooked mussels exposed to 5 and 25 nm TiO_2_NPs, respectively. These high bioaccessibility ratios agreed with those of total Ti. The results indicate that all the Ti (ionic and nanoparticulate) is bioaccessible after gastrointestinal digestion.

The total concentrations of Ag and Ti in the digests and bioaccessible fractions, and the concentrations of Ag and Ti as NPs in the extracts and bioaccessible fractions have been plotted in a heatmap (supplementary information, Fig. 1S), where it can be observed the great variability of the concentration of titanium in the different types of seaweed and fractions.

### Total Ag and AgNP cellular transport

The bioavailability of total Ag and AgNPs was investigated in raw and cooked seaweed samples and cooked mussels exposed to AgNPs, studying the transport through the Caco-2 cell monolayer. Table 3 shows the results of total Ag and AgNP cellular transport for 1 h through the Caco-2 cell membrane. Total Ag concentrations in the basolateral fractions were lower than the LOD (0.05 µg g^−1^). Therefore, it can only be deduced that the transport of total Ag must be less than 23.4 and 13.2% for raw and cooked *Palmaria palmata*, respectively, less than 3.0% for raw and cooked Ulva, and less than 1.0% for cooked mussels. On the other hand, the concentrations of Ag as NPs quantified in the basal fractions were also very low, and the percentage of transported NPs were all lower than 0.3%.

**Table 3:** Total Ag and AgNP cellular transport ratios

	Total Ag			AgNPs						
	[Ag] apical (µg g^−1^)	[Ag] Basal/ µg g^−1^	Transport (%)	[AgNPs] apical (part g^−1^)	Apical size (nm)	[AgNPs] basal (part g^−1^)	Basal size (nm)	[Ag] as NPs apical (µg g^−1^)	[Ag] as NPs basal (µg g^−1^)	Transport (%)
Raw Palmaria	0.21	< 0.05	< 23.8	4.39 × 10^8^ ± 6.24 × 10^7^	31 ± 1	5.38 × 10^5^ ± 8.90 × 10^4^	29 ± 2	0.08 ± 0.01	5.35 × 10^–5^ ± 1.97 × 10^–5^	0.1
Cooked Palmaria	0.38	< 0.05	< 13.2	7.09 × 10^7^ ± 6.14 × 10^6^	40 ± 1	5.16 × 10^5^	26 ± 1	0.02 ± 0.01	5.16 × 10^–5^	0.2
Raw Ulva	1.73	< 0.05	< 2.9	3.11 × 10^8^ ± 3.38 × 10^7^	38 ± 1	6.49 × 10^5^ ± 2.43 × 10^5^	27 ± 2	0.10 ± 0.01	7.01 × 10^–5^ ± 6.83 × 10^–6^	0.1
Cooked Ulva	1.69	< 0.05	< 3.0	1.16 × 10^8^ ± 1.25 × 10^6^	46 ± 2	3.08 × 10^6^	22 ± 1	0.06 ± 0.01	1.75 × 10^–4^	0.3
Cooked Mytilus edulis	4.79	< 0.05	< 1.0	8.62 × 10^7^ ± 2.31 × 10^7^	45 ± 1	1.19 × 10^6^ ± 6.90 × 10^5^	21 ± 2	0.04 ± 0.01	6.07 × 10^–5^ ± 2.34 × 10^–5^	0.1

These results are in agreement with those obtained by Abdelkhaliq et al. [35] who evaluated the impact of in vitro digestion on gastrointestinal fate and uptake of AgNPs with different surface modifications through the coculture medium Caco-2/HT29-MTX (human epithelial colorectal adenocarcinoma/human colon adenocarcinoma mucus-secreting cells). These authors reported cellular transports lower than 0.1% for total Ag and AgNPs [35].

### Total Ti and TiO_2_NP cellular transport

The bioavailabilities of total Ti and TiO_2_NPs were also determined in the cooked and raw seaweed samples and the cooked mussels exposed to 25 or 5 nm TiO_2_NPs, using the intestinal epithelium model.

Table 4 shows the results for total Ti and Ti as NPs cellular transport through the Caco-2 cell membrane. Since the Ti concentration as NPs in the alkaline extracts and bioaccessible fractions of cooked and raw *Palmaria palmata* exposed to 5 nm TiO_2_NPs were < LOD, the cellular transport was studied only in the cooked and raw *Palmaria palmata* exposed to 25 nm TiO_2_NPs. Ti was not detected in the basal fraction of raw *Palmaria palmata* (transport < 0.3%), but the cooked *Palmaria palmata* achieved a Caco-2 cellular transport ratio of 3.1%. However, cell transport was close to 0 for Ti as NPs.

**Table 4:** Total Ti and TiO_2_NP cellular transport ratios

	Total Ti			TiO_2_NPs						
	[Ti] apical (µg g^−1^)	[Ti] basal (µg g^−1^)	Transport (%)	[TiO_2_NPs] apical (part g^−1^)	Apical size (nm)	[TiO_2_NPs] basal (part g^−1^)	Basal size (nm)	[Ti] as NPs apical (µg g^−1^)	[Ti] as NPs basal (µg g^−1^)	Transport (%)
Raw Palmaria 25	6.54	< 0.02	< 0.3	1.19 × 10^9^ ± 1.43 × 10^8^	115 ± 5	6.54 × 10^5^ ± 1.10 × 10^5^	111 ± 7	2.40 ± 0.13	0.001 ± 0.0002	0
Cooked Palmaria 25	2.79	0.09 ± 0.06	3.1	1.31 × 10^9^ ± 3.36 × 10^7^	120 ± 14	6.60 × 10^6^	51 ± 1	3.02 ± 0.92	0.001 ± 0.0003	0
Raw Palmaria 5	< 0.02	< 0.02		-	-	-	-	-	-	
Cooked Palmaria 5	< 0.02	< 0.02		-	-	-	-	-	-	
Raw Ulva 25	9.46	0.09	0.9	1.68 × 10^9^ ± 3.36 × 10^7^	105 ± 1	2.53 × 10^6^ ± 4.54 × 10^6^	143 ± 31	2.61 ± 0.05	0.011 ± 0.007	0.4
Cooked Ulva 25	4.32	0.12 ± 0.02	2.7	1.55 × 10^9^ ± 4.79 × 10^7^	106 ± 1	4.57 × 10^6^	60 ± 1	2.46 ± 0.03	-	
Raw Ulva 5	6.46	0.07	1.1	1.84 × 10^9^ ± 3.96 × 10^7^	89 ± 3	9.38 × 10^5^ ± 2.51 × 10^5^	114 ± 18	1.72 ± 0.17	0.002 ± 0.001	0.1
Cooked Ulva 5	4.19	0.07	1.6	4.76 × 10^8^ ± 4.08 × 10^6^	134 ± 1	-	-	1.51 ± 0.01	-	
Cooked Mytilus edulis 25	0.72	0.05	7.4	8.12 × 10^8^ ± 1.82 × 10^7^	75 ± 1	-	-	0.45 ± 0.01	-	
Cooked Mytilus edulis 5	1.48	< 0.02	< 1.4	2.36 × 10^8^ ± 1.15 × 10^7^	116 ± 2	-	-	0.55 ± 0.05	-	

- indicates “ < LOD

In the case of raw and cooked *Ulva* sp. exposed to 25 nm TiO_2_NPs, the total cellular transport ratios were between 0.9 and 2.7%, respectively, and in the case of raw and cooked *Ulva* sp. exposed to 5 nm TiO_2_NPs total Ti cellular transport ratios for Ti as NPs were between 1.1 and 1.6%, respectively. However, the cellular transport ratios for Ti as NPs were lower than 0.4% for raw *Ulva sp.* and both particle sizes.

The highest total Ti cellular transport ratio was found in the cooked *Mytilus edulis* exposed to 25 nm TiO_2_NPs, reaching a cellular transport ratio of 7.4%. On the other hand, the total content of Ti in the basolateral chamber for *Mytilus edulis* exposed to 5 nm TiO_2_NPs was lower than LOD (0.02 µg g^−1^), which implies a cellular transport of less than 1.4%.

The results obtained in this study indicate that although the bioaccessibility ratios for total Ti and Ti as NPs from seaweed and mussels were very high (even 100% in the case of mussels), total Ti and Ti as NPs transport ratios were in all cases lower than 10%. Then, less than 7.4% of total Ti can cross the epithelium and reach the bloodstream.

### AgNPs and TiO_2_NPs in the Caco-2 cell monolayer

Since Ag and Ti are not highly transported from the apical to the basolateral chamber in one hour of incubation, the presence of NPs in the cell membrane was studied using SC-ICP-MS.

**Fig. 3:**
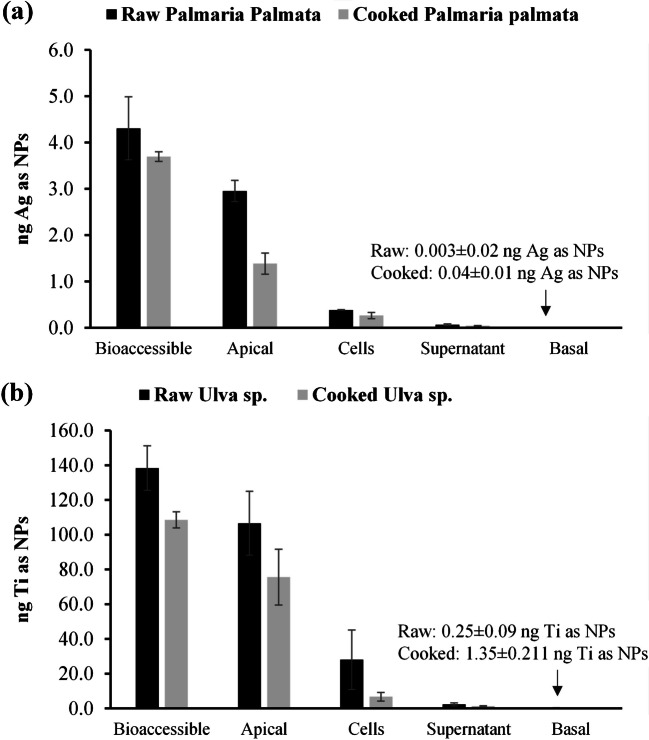
Mass balance in the cellular transport of **a** Ag as NPs in exposed raw and cooked *Palmaria palmata* and **b** Ti as NPs in exposed raw and cooked *Ulva* sp

Raw and cooked *Palmaria* exposed to AgNPs and raw and cooked *Ulva* exposed to 25 nm TiO_2_NPs were used for the in vitro digestion and cellular transport. The Caco-2 cell monolayers were analyzed by SC-ICP-MS to verify the presence of AgNPs and TiO_2_NPs. The bioaccessible, apical, and basal fractions and the supernatant obtained from the centrifugated cells were analyzed by SP-ICP-MS to carry out a mass balance. The SP-ICP-MS and SC-ICP-MS results were transformed as the mass of the element. Figure 3a shows the mass balance for raw and cooked *Palmaria palmata* exposed to AgNPs, and Fig. 3b shows the mass balance for raw and cooked *Ulva* sp. exposed to 25 nm TiO_2_NPs.

The results for the mass balance are also shown in Table S5 (see supplementary information). The results show that most of the mass of nanoparticles remained in the apical fraction, but part of them was detected in the cells and in the supernatant of cells. The Ag mass balance for nanoparticles in raw and cooked seaweed showed a loss of 21% and 54% of AgNP mass during the process, respectively. The mass balance for Ti showed a loss of 1% and 23% in raw and cooked seaweed of the mass of Ti as NPs during the process, respectively. This loss of nanoparticle mass in the SP-ICP-MS analysis can be attributed to the fraction of the nanoparticles with sizes smaller than the LOD.

The mass percentages of Ag as NPs in the Caco-2 cell monolayer (cells + supernatant) were 11 and 8% of the bioaccessible fraction in the raw and cooked *Palmaria palmata*, respectively (where 9% and 7% of Ag mass corresponded to NPs internalized in the cells). On the other hand, the mass percentages of Ti as NPs in the Caco-2 cell monolayer were 22 and 7% for raw and cooked *Ulva* sp., respectively (where 20% and 6% of Ti as NPs corresponded to NPs internalized in the cells).

This current research work shows the bioaccessibility and bioavailability results obtained from raw and cooked seaweed and cooked mussels exposed to 1.0 mg L^−1^ of AgNPs (15 nm) or TiO_2_NPs (25 or 5 nm) for long times (28 days) and submitted to an in vitro digestion process and cellular transport through Caco-2 cell monolayers. Although no research works were found in the literature that calculated the bioavailability of seaweed and mussel samples exposed to NPs, Taboada-López et al. [8] studied the bioaccessibility and bioavailability of unexposed raw mussels samples. The bioaccessibility results for cooked mussels exposed to PVP-AgNPs differ from those reported by Taboada-López et al. [8], where no total Ag and AgNPs had been detected in their bioaccessible fractions from raw unexposed mussel samples, maybe due to the low amount of Ag in the mussels. However, they reported bioaccessibility ratios of 28 ± 7 and 21 ± 3% for total Ti and Ti as NPs, respectively. These percentages differ from the bioaccessibility ratios obtained in this study (100% for total Ti and Ti as NPs for cooked mussels exposed to 25 nm TiO_2_NPs and 100 and 76% for total Ti and Ti as NPs for cooked mussels exposed to 5 mm TiO_2_NPs, respectively). On the other hand, the cellular transport reported by Taboada-López et al. [8] was 40% for total Ti in raw mussels, value higher than the 7.4% cellular transport achieved in the present study for total Ti in cooked mussels exposed to 25 nm TiO_2_NPs. Song et al. [19] achieved insignificant cellular transports for native TiO_2_NPs through the Caco-2 monolayer. However, Koeneman et al. [23] observed a cellular transport of TiO_2_NPs of 14.4% after exposure to 100 μg mL^−1^ of TiO_2_NPs. On the other hand, Imai et al. [14] observed that the cellular transport through the Caco-2 cell monolayer in apical fractions treated with AgNPs depends on the size of the particles and the time of exposure of AgNPs to the cellular medium. These authors observed that the smallest AgNPs pass through the cell membrane more than large ones, and cellular transport increases with the incubation time [14].

These contradictory results come from the use of the different culture conditions, the study of different matrices exposed or not to NPs, and the study of AgNPs and TiO_2_NPs with different sizes and surface properties [19]. Therefore, more studies are needed to elucidate the AgNP and TiO_2_NP bioavailability patterns as a function of the sizes, coating, types of species exposed, and culture conditions.

## Conclusions

Raw and cooked seaweed and cooked mussels exposed to 15 nm PVP-AgNPs, 25 nm or 5 nm citrate TiO_2_NPs were submitted to in vitro gastrointestinal digestions for bioaccessibility determinations. Caco-2 cells simulating the epithelial intestine were selected to perform the bioavailability experiments. Bioaccessibility and bioavailability were determined after quantification of total Ag and Ti and AgNPs and TiO_2_NPs using ICP-MS and SP-ICP-MS, respectively. The bioaccessibility and cellular transport ratios were calculated for the samples, and AgNPs and TiO_2_NPs internalized in the Caco-2 cells were also determined by SC-ICP-MS.

Total Ag and AgNP bioaccessibility ratios in seaweed were between 20 and 42% and between 22 and 97%, respectively. Total Ag and AgNP bioaccessibility ratios in mussels were 100% and 18%, respectively. The decrease in the percentages for mussels could indicate that the AgNP fraction is less accessible than the ionic Ag. On the other hand, total Ti and TiO_2_NP bioaccessibility ratios for seaweed were between 15 and 78% and between 17 and 81%, respectively. In the case of mussels, total Ti and TiO_2_NP bioaccessibility ratios were 100% and between 76 and 100%, respectively.

Nanoparticle cellular transports were in all cases less than 1%. On the other hand, the mass percentages of Ag as NPs and Ti as NPs in the Caco-2 cells for raw and cooked seaweed were 9 and 7% and 20% and 6%, respectively. These results confirm a small transport through the Caco-2 cells for 1 h incubation under the proposed experimental conditions. However, even if the percentage of transport is very low, the continued consumption of AgNPs and TiO_2_NPs may increase the risk throughout an individual’s lifetime.

## Supplementary Information

Below is the link to the electronic supplementary material.

ESM 1 (DOCX 104 KB)

## Data Availability

Data is provided within the manuscript or supplementary information files. Other data can be shared upon request.
