# Supplementary material for: Bioaccessibility and cellular transport study of silver and titanium dioxide nanoparticles from exposed seaweed and mussels using Caco-2 cells
Source: Mikrochim Acta. 2025 Mar 8;192(4):216. doi: 10.1007/s00604-025-07066-4 (PMC11890240; doi:10.1007/s00604-025-07066-4)
Supplement: Supplementary file 1 — (DOCX 104 KB) [file 604_2025_7066_MOESM1_ESM.docx]

**Supplementary information**

**Bioaccessibility and cellular transport study of silver and titanium dioxide nanoparticles from exposed seaweed and mussels using Caco-2 cells**

**Juan José López-Mayán^1.^, Raquel Domínguez-González^1.^, María Carmen Barciela-Alonso^1.^, Elena Peña-Vázquez^1. *^, Antonio Moreda-Piñeiro^1.^, Pablo Taboada-Antelo^2.^, and Pilar Bermejo-Barrera^1.^**

^1.^ Trace Element, Spectroscopy and Speciation Group (GETEE), Instituto de Materiais (iMATUS), Faculty of Chemistry, University of Santiago de Compostela, Av. das Ciencias, s/n 15782. Spain

^2.^ Colloids and Polymer Physics Group, Instituto de Materiais (iMATUS), Department of Particle Physics, Faculty of Physics, Universidade de Santiago de Compostela, Rúa Xosé María Súarez Núñez, s/n. E15782, Santiago de Compostela, Spain

Email corresponding author: [elenamaria.pena@usc.es](mailto:elenamaria.pena@usc.es)

**Table of contents**

**Instrumentation___________________________________________________Page 3**

**Standard, reagents and materials_____________________________________Page 4**

**Characterization of PVP-AgNPs and TiO_2_-citrate NPs___________________Page 6**

**Sample digestion and extraction procedures (Table S1)__________________Page 7**

**In vitro digestion procedures_________________________________________Page 8**

***Bioaccessibility assay***

***Cellular transport assay***

**Cell viability assay________________________________________________Page 10**

**Ag and Ti determination by ICP-MS_________________________________Page 12**

**AgNPs and TiO_2_NPs determination by SP-ICP-MS and SC-ICP-MS_______Page 12**

**Table S2_________________________________________________________Page 14**

**Table S3_________________________________________________________Page 15**

**Table S4_________________________________________________________Page 16**

**Table S5_________________________________________________________Page 17**

**Fig. S1__________________________________________________________Page 18**

**Instrumentation**

A NexION^®^ 2000 inductively coupled plasma mass spectrometer from Perkin Elmer (Waltham, MA, USA) was used for Ag and Ti determination using the Syngistix™ 2.5 software from Perkin Elmer. The ICP-MS working in single-particle mode (SP-ICP-MS) with the Syngistix™ Nano Application 2.5 software, from Perkin Elmer, was used for the determination of AgNPs and TiO_2_NPs content and size distribution. The single-cell Micro DX autosampler from PerkinElmer (Waltham, MA, USA) with the Asperon^TM^ spray chamber allows to work in single-cell mode (SC-ICP-MS), with the Syngistix™ Single Cell Application Software, for determination of AgNPs and TiO_2_NPs internalized in cells. The Ethos Plus microwave lab station from Milestone (Bergamo, Italy) was used for sample acid digestion previous to the total determination of the total content of the elements.

The ultrasound probe VibraCell^TM^ VCX 130 V from Sonics (Newtown, CT, USA) and the ultrasound bath from VWR (Barcelona, Spain) were used for enzymatic and alkaline extractions, respectively. The Boxcult temperature-controlled incubation chamber from Stuart Scientific (Surrey, UK), equipped with a Rotabit orbital-rocking platform shaker from J.P. Selecta (Barcelona, Spain) was employed for enzymatic extractions and *in vitro* digestions. The pH meter from Crison (Barcelona, Spain) was also used for sample preparation.

The UV-Vis spectrometer 689 from Bio-Rad (Hercules, CA, USA) with microplate absorbance reads at 450 nm, was used for the cell viability assessment. The Semi-Micro Osmometer K-7400S form KNAUER (Berlin, Germany) was used for osmolarity adjustments of bioaccessible fractions. The SMH-100 laminar flow cupboard from Telstar (Tarrasa, Spain) was employed to perform the cellular transport experiment. The icoMed incubator from Memmert, (Schwabach, Germany) with a controlled atmosphere was employed for cell culturing. The Electrical Resistance System millicell ERS-2 from Millipore Co. (Massachusetts, USA) and the fluorimeter FLUOstar Omega with a microplate reader from BMG Labtech (Leicester, UK) were used for transepithelial electrical resistance (TEER) and Lucifer Yellow (LY) measurements, respectively. Other instrumentations employed were a Reax top vortex vibrational shaker from Heidolph (Schwabach, Germany), and a heating bath from J.P. Selecta (Barcelona, Spain).

**Standards, reagents, and materials**

Ultrapure water (18 MΩ cm of resistivity), obtained from a Milli-Q^®^ IQ7003 from Millipore Co. (Bedford, MA, USA), 69 % (w/v) SUPRAPUR^®^ nitric acid from Sigma Aldrich, (Darmstadt, Germany), and 33 % (w/v) hydrogen peroxide from ACS, ISO, AppliChem Panreac (Barcelona, Spain) were used for microwave-assisted acid performance. Pancreatin from porcine pancreas and lipase from Candida rugose, both from Sigma Aldrich, a physiological buffer of NaH_2_PO_4_·H_2_O and NaOH both from Merck (Darmstadt, Germany), a mixture of enzymes obtained from *Rhizopus* sp. (Macerozyme^®^ R-10) from Merck (Darmstadt, Germany), a buffer of citric acid for analysis ACS, from Panreac (Barcelona, Spain), and trisodium citrate di-hydrated from Merck were used for enzymatic extractions. For alkaline extractions, 25 % (v/v) tetramethylammonium hydroxide (TMAH) from Merck was employed. P-7000 pepsin from porcine gastric mucosa, P-1750 pancreatin from porcine pancreas, and bile salts (50 % sodium cholate, 50 % deoxycholate) –all from Sigma Aldrich, 96 % sodium taurocholate hydrated from Alfa Aesar (Kandel, Germany), 37 % hydrochloric acid from Merck, sodium hydrogen carbonate from Panreac, D(+)-glucose and sodium chloride both from Merck, were used for *in vitro* digestions. For cellular transport, Lucifer yellow (LY) from Sigma Aldrich, Caco-2 cells cultured at standard conditions (5 % CO_2_ at 37 ºC) in Dulbecco’s Modified Eagle Medium (DMEM), supplemented with 10 % (v/v) fetal bovine serum (FBS) and 1 % (v/v) penicillin/streptomycin, 1 mM sodium pyruvate, 0.1 mM nonessential amino acids (NEAAs), and Transwell^®^ permeable supports of 24 mm insert, 6 well plates and 0.4 µm polyester membrane, from Corning (Kennebunk, ME, USA) were used. A CCK-8 cytotoxicity kit (Sigma Aldrich) was employed for the cell viability assay. Trypsin-EDTA solution, fetal bovine serum (both from Sigma Aldrich), and Cryopres dimethyl sulfoxide from MP Biomedicals, LLC, were employed to lift the cells from the transwell and freeze them.

Other reagents, standards, and materials include an ionic Ag stock solution of 1000 mg L^‑1^ in 2 % HNO_3_ and Ti 1000 mg L^-1^ H_2_O/0.24 % F^-^ both from Perkin Elmer, PEG-COOH gold nanospheres in aqueous 1 mM citrate suspension of 49.6 nm of 9.89×10^6^ particles mL^-1^ from NanoComposix (San Diego, CA, USA), citrate cilver nanospheres, NanoXact^TM^ standards of 20, 40, and 60 nm from nanoComposix (San Diego, CA, USA), a TiO_2_NPs suspension (mixture of rutile and anatase, 99.5 %, <150 nm, 40wt. % in water) (Sigma-Aldrich), Rh stock solution of 1000 mg L^-1^ in 2 % HCl (Perkin Elmer), NexION Setup Solution of 1.0 µg L^-1^ of Be, Ce, Fe, In, Li, Mg, Pb, and U in 1 % of HNO_3_ (Perkin Elmer), high purity 99.999 % Ar, He, and NH_3_ all from Nippon Gases (Madrid, Spain), 1% (v/v) glycerol ACS, EMSURE^®^ (Merck), 1 % (v/v) phosphate-buffered saline (PBS) (Thermo Fisher, Dublin, Ireland), and Minisart NML hydrophilic non-sterile 5.0 µm filter disks (Sartorius, Goettingen, Germany) were also employed.

**Characterization of PVP-AgNPs and TiO_2_-citrate NPs**

The nanoparticles used in this study were characterized in ultrapure water by Araújo et al. (2022) [1] by TEM (primary size of 29.0 ± 0.4 nm for 25 nm citrate-TiO_2_ NPs, and 24.0 ± 0.5 nm for PVP-AgNPs, values higher than those provided by the supplier), and by dynamic light scattering in ultrapure and artificial seawater (35 ppm salinity). AgNPs were stable while the TiO_2_NPs formed much bigger aggregates in seawater (where the surface charge was close to 0, zeta potential -3 mV). The size of 5 nm citrate-TiO_2_NPs was calculated by X-ray diffraction obtaining a size of 8.6 nm. The internalization of the nanoparticles of citrate-TiO_2_NPs could be observed in Ulva sp. by electron microscopy combined with energy dispersive analysis (EDX) [2], while PVP-AgNPs underwent sulphidation/formation of a sulphur rich corona during the process [3].

[1] Araújo MJ, Sousa ML, Fonseca E, Felpeto AB, Martins JC, Vázquez M, Mallo N, Rodriguez-Lorenzo L, Quarato M, Pinheiro I, Turkina MV, López-Mayán JJ, Peña-Vázquez E, Barciela-Alonso MC, Spuch-Calvar M, Oliveira M, Bermejo-Barrera P, Cabaleiro S, Espiña B, Vasconcelos V, Campos A, (2022) Proteomics reveals multiple effects of titanium dioxide and silver nanoparticles in the metabolism of turbot, Scophthalmus maximus. Chemosphere 308:136110.

<https://doi.org/10.1016/j.chemosphere.2022.136110>.

[2] López-Mayán JJ, Álvarez-Fernández B, Peña-Vázquez E, Barciela-Alonso MC, Moreda-Piñeiro A, Maguire J, Mackey M, Quarato M, Pinheiro I, Espiña B, Rodríguez-Lorenzo L, Bermejo-Barrera P, (2023) Bioaccumulation of titanium dioxide nanoparticles in green (Ulva sp.) and red (Palmaria palmata) seaweed. Microchim Acta 190:287. <http://dx.doi.org/10.1007/s00604-023-05849-1>.

[3] Quarato M, Rodriguez Lorenzo L, Pinheiro I, López-Mayán JJ, Mackey M, Moreda-Piñeiro A, Spuch-Calvar M, Maguire J, Bermejo-Barrera P, Correa-Duarte MA, Espiña B, (2024) Bioaccumulation, biodistribution, and transformation of polyvinylpyrrolidone-coated silver nanoparticles in edible seaweeds, Sci Total Environ 949:174914. <https://doi.org/10.1016/j.scitotenv.2024.174914>

**Sample digestion and extraction procedures**

**Table S1.** Extraction conditions of AgNPs and TiO_2_NPs from mussel and seaweed

| **Mussels’ enzymatic extraction** | | |
| --- | --- | --- |
| Parameter | Ag | Ti |
| Mass of sample | 1.0000 g | 1.0000 g |
| Pancreatin/lipase concentration | 3.0 g L^-1^ | 8.0 g L^-1^ |
| Volume of enzymatic solution | 7.5 mL | 7.5 mL |
| Incubation time | 12 h | 12 h |
| Temperature of incubation | 37 ºC | 37 ºC |
| Incubation chamber speed | 150 rpm | 200 rpm |
| **Seaweed’s enzymatic extraction** | | |
| **Parameter** | **Ag** | |
| Mass of sample | 0.0500 g | |
| Macerozyme R-10^®^ concentration | 25 g L^-1^ | |
| Volume of enzymatic solution | 9 mL | |
| Probe sonication time | 2.5 min (pulses: 1s on, 1s off) | |
| Incubation time | 6 h | |
| Temperature of incubation | 37 ºC | |
| Incubation chamber speed | 150 rpm | |
| **Seaweed’s alkaline extraction** | | |
| **Parameter** | **Ti** | |
| Mass of sample | 1.0000 g | |
| Final volume of 2.5 % (v v^-1^) TMAH | 10 mL | |
| Sonication time | 2 h (continuous) | |

**In vitro digestion procedures**

***Bioaccesibility assay***

The human gastrointestinal *in vitro* digestion was carried out in two successive steps simulating the gastric and intestinal conditions. To perform the gastric step, 0.5000 g of homogenized samples (raw or cooked) were mixed with 10 mL of ultrapure water. After waiting a few minutes for pH stabilization (pH= 2.0 with 0.1 M HCl), and adding 75 µL of gastric solution (160 g L^‑1^ of pepsin in 0.1M HCl), the Erlermeyer flasks were covered with Parafilm^®^ and incubated in the Boxcult chamber at 37 ºC and 150 rpm for 2 hours. The gastric extracts were then introduced in an ice bath to stop the enzymatic activity. The intestinal step starts by adjusting the pH of the gastric fraction to 7.0 (0.1 M NaOH), and the addition of the intestinal simulating solution (2.5 mL of a mixture of 4 g L^-1^ of pancreatin and 2.58 g L^-1^ of sodium taurocholate hydrated in 0.1M of NaH_2_CO_3_). The Erlenmeyer flasks were covered again and submitted to incubation at 37 ºC and 150 rpm, for 2 hours. The intestinal extracts were cooled in an ice bath to stop the enzymatic activity. Finally, the intestinal extracts were filtered with 5.0 µm filter disks, and the bioaccessible fractions were stored in polypropylene tubes at -18 ºC until use. Three replicates of samples and two blanks were used for each *in vitro* digestion batch.

***Cellular transport assay***

The bioaccessible fractions (around 10 mL) obtained after the *in vitro* digestion were heated at 90 ºC for 5 min to decompose the residual gastrointestinal enzymes. Afterwards, the fractions were divided into two tubes containing 5 mL of the bioaccessible fraction, and 50 µL of 100 g L^-1^ of D(+)-glucose solution was added. The osmolarity of each fraction was adjusted with the addition of 5 M NaCl solution dropwise to obtain an osmolarity between 290±10 mOsm kg^-1^, required for cell viability. Finally, 100 µL of 5 mM of Lucifer yellow (LY) was added to each bioaccessible fraction as a fluorescent marker, and maintained at 37 ºC in a heating bath before their deposition in the Transwells^®^ containing the Caco-2 monolayer.

The Caco-2 cells line was maintained in Dulbecco´s Modified Eagle Medium (DMEM) supplemented with 10 % (v/v) fetal bovine serum (FBS), 1 mM sodium pyruvate, 1 mM non-essential amino acids (NEAAs), and 1 % (v/v) penicillin/streptomycin. The cells were incubated at standard conditions at 37 ºC with 95 % of relative humidity, with a 5 % of CO_2_ flow. Every two or three days the medium was replaced, reaching 80 % of confluence. Then, the cells were detached with 0.5 g L^-1^ trypsin and resuspended in DMEM. Finally, the cells were seeded in the polyester membranes of the 6-well Transwells^®^ with an initial cell concentration of 5×10^4^ cells cm^-2^. The Transwells^®^ are divided into the apical compartment (upper) which simulates the intestinal lumen, and the basolateral compartment (lower) which simulates the serous cavity. Both parts are separated by the polyester membrane where the monolayer of Caco-2 cells grows. Cells were deposited apically (1.5 mL) and the nutrient medium was deposited basolaterally (2 mL of DMEM). The transwells containing the cells were incubated in the chamber at 37 ºC with 95 % of humidity and 5 % of CO_2_ flow. The basal DMEM was replaced every three days. The monolayer was allowed to grow until TEER values around 150 mΩ cm^2^ were obtained, and no holes were observed in the monolayer under the microscope.

Once the monolayer was formed the transport assays were performed adding 1.5 mL of the bioaccessible fraction into the apical compartment and 2 mL of HBSS in the basolateral chamber. The Transwell^®^ containing the bioaccessible fractions were incubated in the same conditions as the Caco-2 cells for one hour. After this time of incubation, the integrity of the monolayer was determined by monitoring the transepithelial electric resistance (TEER) and by assessing the permeability of the monolayer to Lucifer Yellow (LY). LY assays measure fluorescence differences between the basal fraction and the initial bioaccessible fraction with LY, where < 2 % of transport of LY through the membrane is necessary to ensure membrane integrity. After the incubation, TEER measurements must be the initial value TEER±25 % to ensure also the cell membrane integrity. The apical and basolateral fractions were collected and stored until their analysis by SP-ICP-MS. The cells adhering to the membrane must be lifted without breaking them, to perform the SC-ICP-MS analysis of the nanoparticles. For that, 1.0 mL of PBS was added to the apical compartment to clean the membrane, and then, the addition of trypsin and incubation for 4 min at 37 ºC, 95 % relative humidity and 5 % CO_2_ flow, was carried out to separate the cells. Finally, the cells were frozen at -18 ºC keeping their integrity using Cryopres dimethyl sulfoxide, until their analysis by SC-ICP-MS. Each bioaccessible fraction was submitted in triplicate to the cellular transport across the Caco-2 cell membrane with their respective blanks.

**Cell viability assay**

The CCK-8 cytotoxicity kit was used to study the viability of the Caco-2 cells membrane after the addition of the bioaccessible fractions containing different salts from the *in vitro* intestinal digestion (bile salts or sodium taurocholate). The use of bile salts might result in the cell monolayer rupture, and then a viability experiment using a purer reagent was assessed.

For the *in vitro* toxicity assay Caco-2 cells were seeded into 96-well plates at 1×10^4^ cells/well. To allow complete cell attachment, cells were incubated for 24 h at 37ºC and 5 % CO_2_ in 100 μL growth medium. The cells in the culture medium were used as the negative control. After 24 h of incubation, 100 μL of a sample with bile salts, a sample with sodium taurocholate and two blanks (one with bile salts and the other with sodium taurocholate) were added into the wells and incubated for 2h, 3h, 4h, and 24h. After incubation, the culture medium was discarded, and the cells were washed with 10 mM PBS (pH=7.4) three times. Then, 100 μL of culture medium containing 10 μL of CCK-8 reagent was added to each well and left for incubation for 1 h. Cell viability was quantified via UV-Vis absorption measurements at 450 nm using a microplate absorbance reader. Cell viability was calculated using Equation (1).

$$Cell viability \left( \% \right)=\frac{\mathrm{Abs}_{\mathrm{sample}}}{\mathrm{Abs}_{\mathrm{control}}} x 100 \left( 1 \right)$$

Where Abs_sample_ is the absorbance at 450 nm for cells incubated with the solutions, and Abs_control_ is the absorbance for control (only cells). Experiments were performed in quadruplicate.

After 2 h, the cells in contact with samples containing sodium taurocholate, bile salts, and the blanks with taurocholate reported viability percentages higher than 80 %, being higher than the viability of the blank with bile salts. A reduction in viability for all the samples was observed after 3 h, being these solutions toxic at 24 h. Sodium taurocholate was the preferred choice over bile salts because it was biocompatible within the first two hours, which would be the maximum duration time allowed for the cellular transport experiments.

**Ag and Ti determination by ICP-MS**

Total Ag and Ti concentrations were determined in raw and cooked seaweed and cooked mussel acid digests, in the bioaccessible fractions, and in the basolateral solutions from the Caco-2 *in vitro* assays, by ICP-MS. Table S2 (a) shows the operational conditions of ICP-MS. Total Ag determination was performed using the standard addition calibration (0-10.0 µg L^-1^) to avoid matrix effects, using ^103^Rh as an internal standard, and Kinetic Electronic Discrimination (KED) mode with He to avoid polyatomic interferences. Total Ti determination was also performed using standard addition calibration (0-15.0 µg L^-1^) and a Dynamic Reaction Cell (DRC) with 1.0 mL min^‑1^ of ammonia to generate the Ti-ammonia cluster of m/z 131, avoiding the potential ^48^Ca interference.

The limits of detection (LOD) and quantification (LOQ) were calculated using the 3σ/m and 10σ/m criteria where σ is the standard deviation of ten measurements of a blank, and m is the slope of the standard addition calibration graph. Table S3 shows the LOD and LOQ for Ag and Ti in seaweed and mussel digestions, bioaccessible fractions, and basolateral fractions after the Caco-2 cellular transport.

**AgNP and TiO_2_NP determination by SP-ICP-MS and SC-ICP-MS**

AgNPs and TiO_2_NPs content and size distributions were determined by SP-ICP-MS. AgNPs and TiO_2_NPs extractions were performed in the pool of samples used for the bioaccessibility and cellular transport assays. AgNPs and TiO_2_NPs were also determined directly in the bioaccessible fractions and the apical and basolateral fractions from the cellular transport through the Caco-2 cell membrane. Table S2 (b) shows the specific instrumental conditions for SP-ICP-MS. The transport efficiency in SP-ICP-MS was automatically assessed by the software after measuring the flow rate, an ionic gold calibration (0-3.0 µg L^-1^), and using the gold nanosphere reference material. Due to the high dilution of the samples (500 to 1000 times) in SP-ICP-MS, external calibrations were performed for Ag (0-5.0 µg L^-1^) and Ti (0-10.0 µg L^-1^). The Syngistix™ Nano Application software also allows working in DRC mode in the case of TiO_2_NPs determination. The enzymatic and alkaline extracts, the bioaccessible, and the apical and basolateral fractions were diluted with 1 % (v/v) of glycerol before SP-ICP-MS analysis. Laborda et al. [34] criteria were used for the LOD determination in SP-ICP-MS mode. Table S4 shows the LOD_number_ and the LOD_size_ of AgNPs and TiO_2_NPs obtained in the enzymatic and alkaline extracts, and the bioaccessible, apical and basolateral fractions.

SC-ICP-MS was used to determine the AgNPs and TiO_2_NPs internalized in the Caco-2 cells. The appropriate dilution of the sample, short dwell times, and a minimum sample uptake rate (10 µL min^-1^) are needed. Table S2 (c) shows the specific operational conditions for SC-ICP-MS. External ionic calibrations (0-3.0 µg L^-1^ and 0-10.0 µg L^-1^ for Ag and Ti), and standards containing AgNPs (20, 40, and 60 nm, 1.0 µg L^-1^) and TiO_2_NPs (150 nm, 8 µg L^-1^) were employed. The LODs have been automatically assessed with the Syngistix™ ICP-MS software, and 5.0±1.4 and 95±11 attograms per cell for Ag and Ti were obtained, respectively.

**Table S2** Operational conditions for (a) ICP-MS, (b) SP-ICP-MS, and (c) SC-ICP-MS

| 1. **Operational conditions ICP-MS** | | |
| --- | --- | --- |
| **Parameter/component** | **Type/mode/value** | |
| Nebulizer | Meinhard CR R^+^ | |
| Nebulizer Chamber | 5 ºC refrigerated glass cyclone chamber with Peltier PC^3X^ | |
| Cone material | Nickel/aluminum | |
| Radiofrequency power | 1600 W | |
| Ar gas flow | 15 L min^-1^ (Plasma) | |
|  | 1.15 L min^-1^ (Nebulizer) | |
|  | 1.2 L min^-1^ (Auxiliary) | |
| Sample loop | 1 mL | |
| Analytes | Ti and Ag | |
| Operation mode | KED for Ag and DRC for Ti | |
| Helium flow rate | 4.5 mL min^-1^ | |
| Ammonia flow rate | 1.0 mL min^-1^ | |
| Integration time | 1000 ms | |
| m/z | ^107^Ag | |
|  | ^131^ [^48^Ti^14^N^1^H(^14^N^1^H_3_)_4_^+^] | |
| Replicates per sample | 3 | |
| Rejection parameter q | 0.2 | |
| 1. **Specific operational conditions for SP-ICP-MS** | | |
| Sample flow rate |  | 0.19-0.23 mL min^-1^ |
| Operation mode |  | Standard for Ag and DRC for Ti |
| Acquisition time Ag / Ti |  | 100s / 60 s |
| Dwell time Ag / Ti |  | 50 µs / 100 µs |
| Number of readings |  | 2,000,000/600,000 |
| Transport efficiency |  | 11.03-12.70 % |
| 1. **Specific operational conditions for SC-ICP-MS** | | |
| Ar gas flow | Nebulizer | 0.35 L min^-1^ |
|  | Makeup | 0.70 L min^-1^ |
| Sample flow rate |  | 10 µL min^-1^ |
| Loop |  | 100 µL |
| Operation mode |  | Standard for Ag and DRC for Ti |
| Acquisition time |  | 100s |
| Dwell time Ag / Ti |  | 50 µs / 100 µs |
| Transport efficiency |  | 40-60 % |

**Table S3** Limits of detection and quantification of total Ag and Ti in the sample digests, bioaccessible, and basolateral fractions after the cellular transport

| **Microwave-assisted acid digestions** | | | | |
| --- | --- | --- | --- | --- |
|  | Seaweed | | Mussels | |
|  | Ag | Ti | Ag | Ti |
| LOD/ µg g^-1^ | 0.01 | 0.06 | 0.01 | 0.05 |
| LOQ/ µg g^-1^ | 0.03 | 0.21 | 0.03 | 0.17 |
| **Bioaccessible fractions** | | | | |
|  | Seaweed and mussels | | | |
|  | Ag | | Ti | |
| LOD/ µg g^-1^ | 0.09 | | 0.28 | |
| LOQ/ µg g^-1^ | 0.29 | | 0.94 | |
| **Basolateral fractions after the Caco-2 cell transport** | | | | |
|  | Seaweed and mussels | | | |
|  | Ag | | Ti | |
| LOD/ µg g^-1^ | 0.05 | | 0.02 | |
| LOQ/ µg g^-1^ | 0.10 | | 0.06 | |

**Table S4** Limits of detection of AgNPs and TiO_2_NPs in sample extracts, bioaccessible, and basolateral fractions

| **Enzymatic extracts from the pool seaweed and mussel samples** | | |
| --- | --- | --- |
|  | AgNPs | TiO_2_NPs |
| LOD_number_/ NPs L^-1^ | 5.60×10^5^ | 1.17×10^6^ |
| LOD_number_/ NPs g^-1^ | 1.01×10^8^ | 7.17×10^7^ |
| LOD_size_ 5σ/ nm | 18 | 29 |
| **Bioaccessible fractions** | | |
|  | AgNPs | TiO_2_NPs |
| LOD_number_/ NPs L^-1^ | 1.41×10^6^ | 3.27×10^6^ |
| LOD_number_/ NPs g^-1^ | 3.67×10^7^ | 9.69×10^7^ |
| LOD_size_ 5σ/ nm | 19 | 28 |
| **Basolateral fractions after Caco-2 cellular transport** | | |
|  | AgNPs | TiO_2_NPs |
| LOD_number_/ NPs L^-1^ | 1.75×10^6^ | 4.17×10^5^ |
| LOD_number_/ NPs g^-1^ | 4.17×10^7^ | 4.86×10^7^ |
| LOD_size_ 5σ/ nm | 19 | 29 |

**Table S5** Mass balance in the cellular transport of Ag as NPs in exposed raw and cooked *Palmaria palmata*, and Ti as NPs in exposed raw and cooked *Ulva* sp

|  | **ng Ag as NPs in Palmaria Palmata** | **ng Ti as NPs in Ulva sp.** |
| --- | --- | --- |
| **Raw seaweed** | | |
| Bioaccessible | 4.31±0.68 | 138.39±12.78 |
| Apical | 2.95±0.93 | 106.60±18.36 |
| Cells | 0.39±0.01 | 28.03±17.12 |
| Supernatant | 0.07±0.02 | 2.29±0.83 |
| Basolateral | 0.003±0.002 | 0.25±0.09 |
| **Cooked seaweed** | | |
| Bioaccessible | 3.70±0.10 | 108.57±4.66 |
| Apical | 1.39±0.23 | 75.60±16.06 |
| Cells | 0.27±0.07 | 6.73±2.48 |
| Supernatant | 0.04±0.01 | 1.35±0.21 |
| Basolateral | 0.002±0.001 | 0.23±0.01 |

**Fig. 1S** Bioaccessibility assay: Heatmap of concentrations (units µg g^-1^)
